# Skeletal muscle Heat shock protein 60 increases after endurance training and induces *peroxisome proliferator-activated receptor gamma coactivator 1 α1* expression

**DOI:** 10.1038/srep19781

**Published:** 2016-01-27

**Authors:** Rosario Barone, Filippo Macaluso, Claudia Sangiorgi, Claudia Campanella, Antonella Marino Gammazza, Viviana Moresi, Dario Coletti, Everly Conway de Macario, Alberto JL Macario, Francesco Cappello, Sergio Adamo, Felicia Farina, Giovanni Zummo, Valentina Di Felice

**Affiliations:** 1 Department of Experimental Biomedicine and Clinical Neurosciences (BioNeC), University of Palermo, Palermo, 90127 Italy; 2 Euro-Mediterranean Institute of Science and Technology (IEMEST), Palermo, 90100, Italy; 3 Department of Anatomical, Histological, Forensic & Orthopaedic Sciences, Section of Histology & Medical Embryology, Sapienza University of Rome, Rome, 00161, Italy; 4 Interuniversity Institute of Myology, Rome, 00161, Italy; 5 University Pierre et Marie Curie Paris, UR4 Aging, Stress, Inflammation, 75005 Paris, France; 6 Department of Microbiology and Immunology, School of Medicine, University of Maryland at Baltimore, IMET, 21201 Baltimore, MD, USA

## Abstract

Heat shock protein 60 (Hsp60) is a chaperone localizing in skeletal muscle mitochondria, whose role is poorly understood. In the present study, the levels of Hsp60 in fibres of the entire posterior group of hindlimb muscles (*gastrocnemius*, *soleus*, and *plantaris*) were evaluated in mice after completing a 6-week endurance training program. The correlation between Hsp60 levels and the expression of four isoforms of peroxisome proliferator-activated receptor gamma coactivator 1 alpha (PGC1α) were investigated only in *soleus*. Short-term overexpression of *hsp60*, achieved by *in vitro* plasmid transfection, was then performed to determine whether this chaperone could have a role in the activation of the expression levels of PGC1α isoforms. The levels of Hsp60 protein were fibre-type specific in the posterior muscles and endurance training increased its content in type I muscle fibers. Concomitantly with the increased levels of Hsp60 released in the blood stream of trained mice, mitochondrial copy number and the expression of three isoforms of PGC1α increased. Overexpressing *hsp60* in cultured myoblasts induced only the expression of *PGC1 1α*, suggesting a correlation between Hsp60 overexpression and PGC1 1 α activation.

The chaperoning system participates in many cellular functions from assisting protein folding and assembling of multimolecular complexes to maintaining the correct shape of enzymes^1^,^2^,^3^,^4^,^5^,^6^,^7^. In addition, extracellular chaperones contribute to the intercommunication between different cells, tissues, and organs^8^,^9^,^10^. It follows that exercise, which requires the participation of various intercommunicating muscle-cell types and intracellular components is most likely dependent on the chaperoning system, or at least some of its components. This is suggested by the presence of various heat shock protein (Hsp)-chaperones in skeletal muscle: sHsp, Hsp60, Hsp70, and Hsp90^11^,^12^.

A major deficiency in our knowledge of the chaperoning system pertains to its physiological distribution in the body and the changes that might occur during functional stages of the various organs. Elucidation of the chaperoning system components distinctive of each cell type in a tissue is necessary to fully understand its physiology and to identify pathological alterations that might be amenable to treatment targeting chaperones. Accordingly, we set out to map Hsp60 in skeletal muscle. We focused on Hsp60 because of its preeminent role in the physiology of mitochondria and the importance of the latter in muscle function and motor activities, and because it has been found extracellularly and in circulation, possibly intercommunicating cells. The three main questions we wanted to answer were: Does exercise induce quantitative changes in Hsp60 and are these changes distinctive of fiber type? If Hsp60 levels were found to be increased during exercise what other pertinent molecules are also increased? Do Hsp60 levels in blood increase associated with increased levels in fibers? We mapped the quantitative distribution of Hsp60 in muscle fibers in relation to function in response to exercise. To deal with the second question we investigated the peroxisome proliferation-activated receptor-γ (PPAR-γ) coactivator-1α (PGC1α). We focused on this molecule because it is a dominant regulator of oxidative metabolism, acting as a transcriptional co-activator of nuclear receptors and other transcription factors regulating mitochondrial biogenesis^13^. The latter biogenesis is a key event in the skeletal muscle adaptation generated by endurance training, leading to an increase in the number of mitochondria^14^,^15^,^16^,^17^.

PGC1α expression in muscle can be induced by a variety of stimuli, among which the contractile activity-mediated increase in adenosine monophosphate (AMP)-activated protein kinase (AMPK) and p38 mitogen-activated protein kinase activation^13^. Four alternative isoforms of PGC1α have been recently described^18^. Interactions between PGC1α and mitochondrial HSPs have never been investigated.

We hypothesized that an increase in Hsp60 should not be the same in all fiber types considering their variety, and should be accompanied by changes in PGC1α and increased mitochondrial numbers. If that were to be the case, then it would be possible to plan experiments to dissect the molecular interactions involving Hsp60 in muscle and, by extension, to think of ways to manipulate this chaperone for improving muscle function in ageing people, and patients with cachexia and skeletal muscle disorders.

To test our assumption, we first performed a comprehensive assessment of Hsp60 levels in sedentary and trained mice, comparing different type of fibers. We demonstrated the activation of mitochondrial biogenesis pathway and the overexpression of three isoforms of PGC1α, recently described in trained skeletal muscle. Moreover, using a short-term overexpression of Hsp60 in cultured myoblasts, we also demonstrated a correlation between Hsp60 overexpression and PGC1 α1 activation.

## Results

### Functional effects of endurance exercise

**Figure 1:**
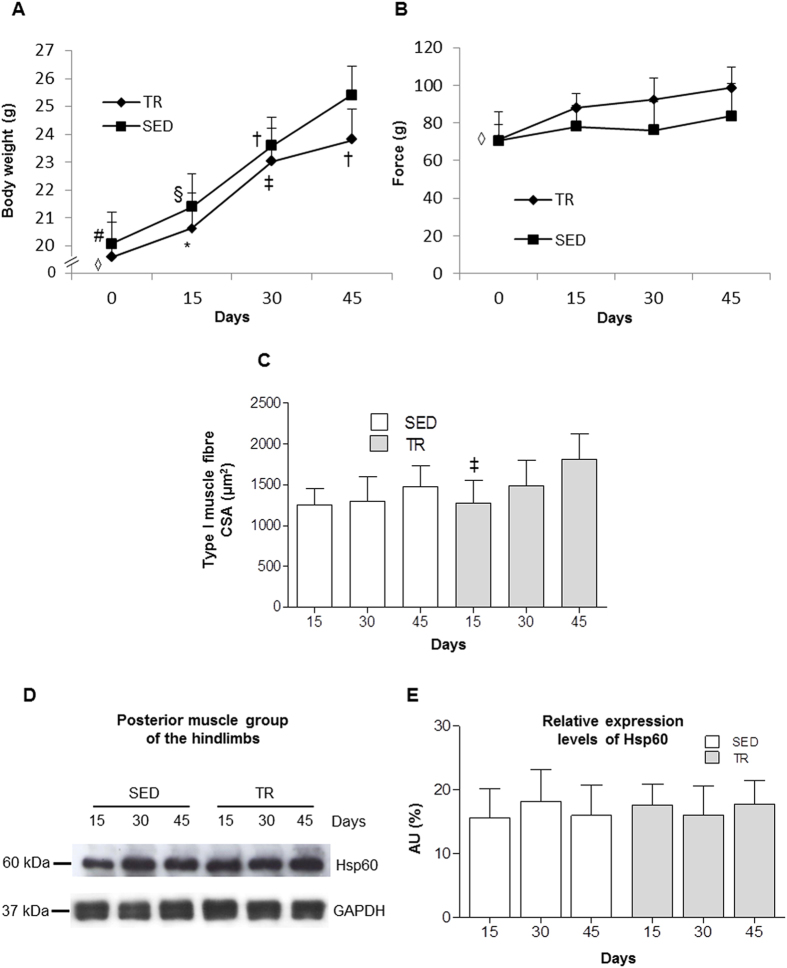
Functional effects of endurance exercise on the body weight, strength, cross-sectional area (CSA) and Hsp60 protein levels in the posterior group of hindlimb muscles. (**A**), changes in the body weight over time. Square (▄) sedentary (SED) mice; Rhombus (♦) trained (TR) mice; horizontal axis, time of training. Data are presented as the means ± SD. # significantly different from SED15 (n = 8), SED30 (n = 8) and SED45 (n = 8) mice (P < 0.001); * significantly different than TR30 (n = 8) and TR45 (n = 8) mice (P < 0.001); § significantly different from SED30 and SED45 mice (P < 0.001); ‡ significantly different from TR45 mice (P < 0.001); † significantly different from SED45 mice (P < 0.05). (**B**) strength of the forelimbs. Square (▄) SED mice; Rhombus (♦) TR mice; horizontal axis, time of training. Data are presented as the means ± SD. ◊ significantly different from TR15 (n = 8), TR30 and TR45 (P < 0.001). (**C**) histogram showing the results for the CSA of type I fibers of the posterior group of hindlimb muscles. An average of 435 fibers was analyzed for each mouse. Bars, groups of mice; open bars, SED mice; shaded bars, TR mice; horizontal axis, time of training. (**D**) representative western blots of Hsp60 (60 kDa) levels in the posterior group of hindlimb muscles (gastrocnemius, soleus, and plantaris) at various time points (n = 3). 60 μg of protein was loaded in each lane; GAPDH (37 kDa) was used as the loading control. (**E**) relative expression levels of Hsp60 from the sedentary and trained groups of mice at various times of training, as shown in the horizontal axis. AU: Arbitrary Unit.

The mice were weighed at the beginning of the experiment and every two weeks thereafter. The mice from both groups, sedentary (SED) and trained (TR), showed a significant increase in body weight during the 45 days of experimentation (*P* < 0.001). Trained groups for 30 and 45 days (TR30 and TR45, respectively) and sedentary groups for 30 and 45 days (SED30 and SED45, respectively) showed a significant increase in the body weight compared to trained groups for 15 days (TR15) and sedentary groups for 15 days (SED15), respectively (*P* < 0.001); while the body weight of TR45 and SED45 mice increased significantly compared to TR30 and SED30 mice, respectively (*P* < 0.001). Moreover, only the mice trained for 45 days underwent a reduced increase in body weight compared to the corresponding sedentary mice (*P* < 0.05) (Fig. 1A).

To confirm that the endurance training induced skeletal muscle modifications and improved muscular fitness, the strength of the mice and the cross section area (CSA) of the type I muscle fibers were measured. The strength results revealed significant increases in both parameters for the trained groups after 15, 30, and 45 days (*P* < 0.001), while no significant differences were detected in the sedentary groups (Fig. 1B).

The values of the CSA increased with age and endurance training induced a further increase in the CSA of the type I fibers, particularly those involved in aerobic activity. Morphometric analysis revealed a significant increase in the CSA of the type I fibers from TR45 mice compared to the TR15 mice (*P* < 0.001) (Fig. 1C). These increases in the strength of the mice and CSA of the type I muscle fibers demonstrated that endurance training induced significant adaptations in skeletal muscle.

### Hsp60 levels in the posterior muscle group of the hindlimbs (bulk analysis)

**Figure 2:**
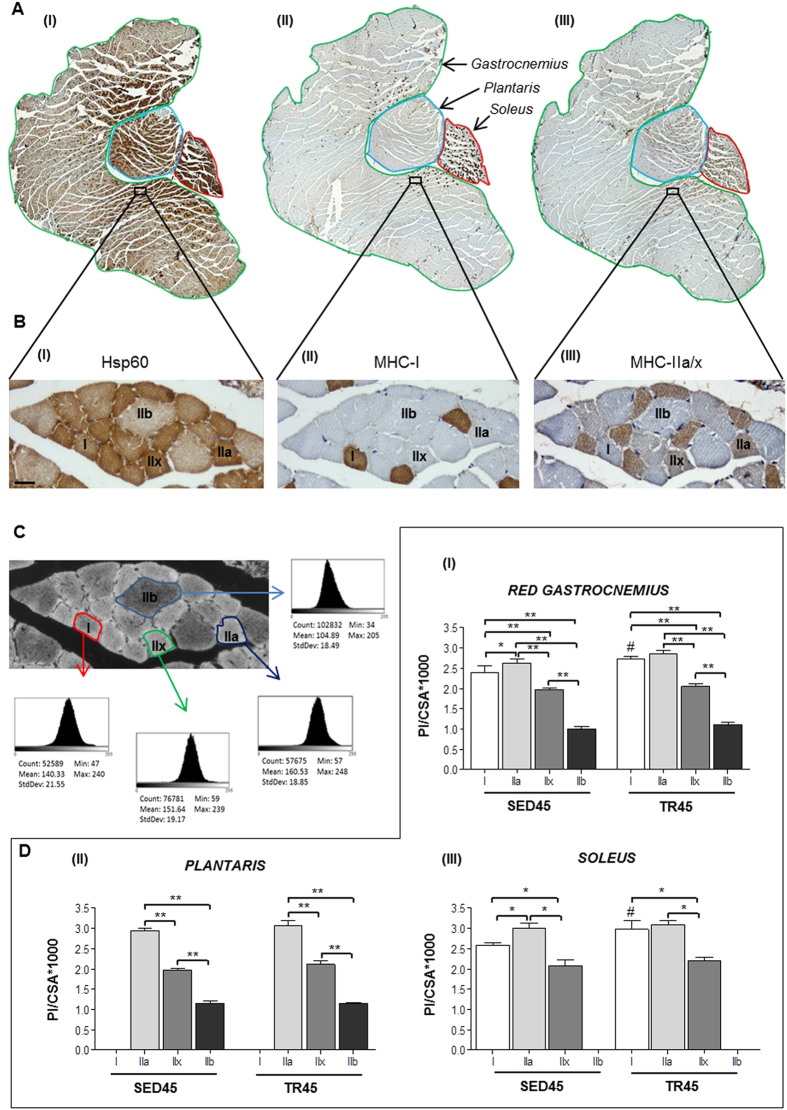
Immunohistochemistry and densitometric analysis of the staining intensity in the posterior group of hindlimb muscles demonstrate that Hsp60 is elevated in type IIa and I muscle fibers, and with endurance training. (**A**) immunohistochemistry for Hsp60 (I), MHC-I (II) and MHC-IIa/x (III) in serial cross-sections of the posterior group of hindlimb muscles, reconstructed by combining multiple images captured at low magnification (10×) (*gastrocnemius*, *soleus*, and *plantaris*, are circled in green, red, and blue, respectively). (**B**) enhanced magnification of the images shown in A. Type IIb fibers are negative to antibodies anti-MHC-I and anti-MHC-IIa/x. Bar 25 μm. (**C**) Representative images used for densitometric analysis of the staining intensity of a cross-section immunostained for Hsp60. The analysis was performed using 5 fields per section, 5 sections per mice (40 μm between sections) and 8 mice per group. An average of 435 fibers was analyzed for each mouse. The acquired RGB image (Bb) was transformed to a greyscale image (32-bit) and then inverted. Representative histograms of the fibers I, IIa, IIb, and IIx are shown in the small framed panels, where 0 corresponds to no positivity and 255 corresponds to maximal positivity. (**D**), the staining intensity (determined with ImageJ 1.41 software) of the fibers was expressed as the mean pixel intensity (PI) normalized to the cross-sectional area (CSA) for the red *gastrocnemius* (I), the *plantaris* (II), and the *soleus* (III). SED45 and TR45 indicate trained and sedentary mice on day 45, respectively. Data are presented as the means ± SD. ^#^significantly different from type I fibers from SED45 mice (P < 0.01), *(P < 0.05), **(P < 0.001).

A western blot analyses of Hsp60 in the entire posterior group of hindlimb muscles (*gastrocnemius*, *soleus*, and *plantaris*) did not reveal any significant difference either within the SED and TR groups at different time points or between the SED and TR groups at each time point (Fig. 1D,E). To evaluate Hsp60 levels in each muscle of the posterior group of hindlimb, cross-sections of the entire muscle group were analyzed by immunohistochemistry. There was an appreciable difference in the levels of Hsp60 between the three muscles that form the posterior group (Fig. 2AI). Moreover, a mosaic-staining pattern was detected in the fibers (Fig. 2BI). All of the muscle fibers of the *soleus* were positive for Hsp60, while only approximately 55% of the fibers of the *plantaris* were positive. The *gastrocnemius* displayed positivity for Hsp60 in the deeper region (red area of the *gastrocnemius*), more specifically in the regions lateral and medial to the *plantaris* (Fig. 2AI).

Immunohistochemistry of Hsp60 (Fig. 2, A_I_ and B_I_), myosin heavy chain (MHC) -I (Fig. 2, A_II_,B_II_) and MHC-IIa/IIx (Fig. 2, A_III_,B_III_) were performed on serial cross-sections to evaluate the levels of Hsp60 in each muscle fiber type. Type IIa fibers were strongly stained with the A4.74 antibody (anti-MHC-IIa/IIx) compared to type IIx fibers (Fig. 2BIII). By examining overlapping serial cross-sections of the same sample stained for MHC-I and MHC-IIa/x, it was possible to identify type IIb fibers because they were unstained. Type I fibers were more abundant in the *soleus* compared to the *plantaris* and the *gastrocnemius*, while type IIa and IIx fibers were more abundant in the latter two muscles. Intermediate hybrid fibers were not taken into consideration in these analyses because of their limited number; only a few fibers per mouse were classified as hybrid.

### Hsp60 levels in various muscle fiber types

**Figure 3:**
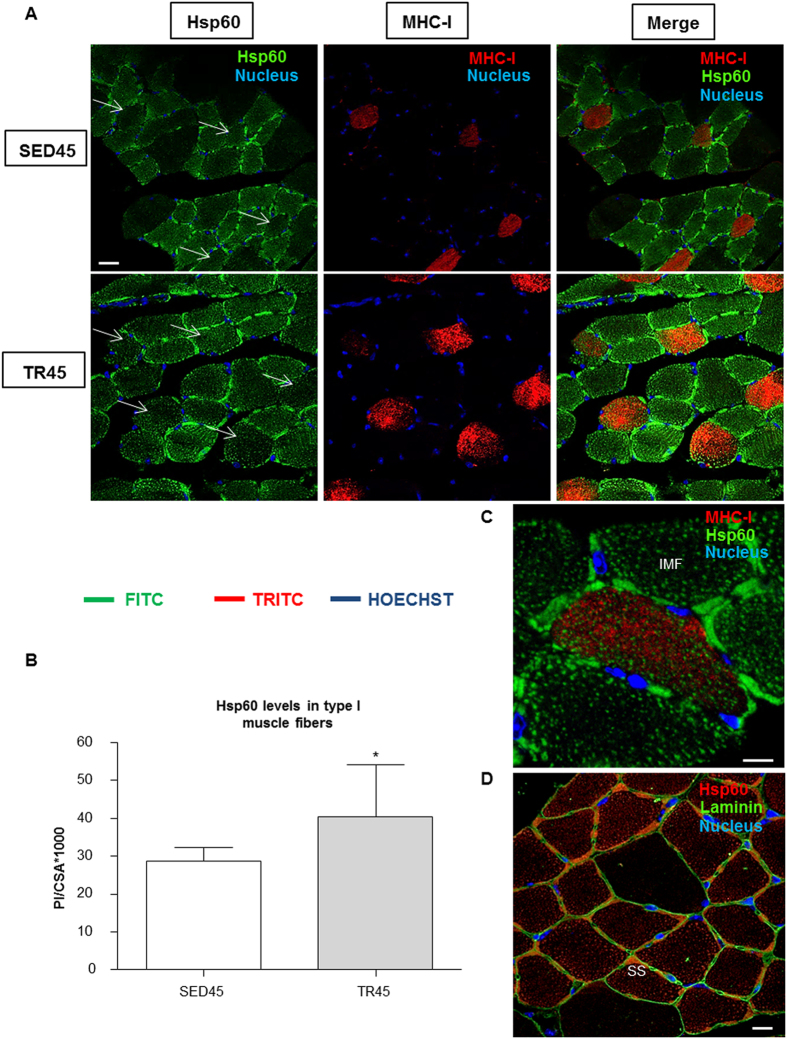
Confocal microscopy analysis further demonstrates that Hsp60 protein levels increase in mice trained for 45 days and shows the localization of Hsp60 in the myofibrillar cell. (**A**) immunofluorescence for Hsp60 and MHC-I of cross-sections of sedentary and trained mice at 45 days (SED45, n = 8, and TR45, n = 8, respectively); the arrows indicate the type I fibers. Bar 25 μm. (**B**) the staining intensity for Hsp60 (bars) of type I fibers was expressed as the mean pixel intensity (PI) normalized to the CSA (cross-sectional area) using the software Leica application suite advanced fluorescences software. Open bar, sedentary (SED45) mice; shaded bar, trained (TR45) mice; both on day 45. Data are presented as the means ± SD. *significantly different from SED45 mice (P < 0.05). (**C**) representative immunofluorescence image revealing that Hsp60 was localized also in inter-myofibrillar mitochondria (IMF). Bar 10 μm. (**D**) representative immunofluorescence image revealing that Hsp60 was localized to the subsarcolemmal (SS) space. Bar 20 μm.

The levels of Hsp60 in each muscle fiber type were analyzed only at 45 days because the overall greatest changes were detected at this time point. Serial cross-sections of muscle from sedentary mice (SED45) analyzed by immunohistochemistry revealed that the Hsp60 protein was more elevated in type IIa fibers than in type I fibers (*P* < 0.05), while in the latter type I fibers Hsp60 was present at higher levels than in type IIx fibers (*P* < 0.05) (Fig. 2D). Type IIb fibers displayed lower levels of Hsp60 than any other fiber type (*P* < 0.05). The same pattern was confirmed in all three muscles analyzed, although the *soleus* and the *plantaris* did not contain sufficient numbers of type IIb and I fibers, respectively, to perform statistical analysis. Endurance exercise training induced a significant increase in the Hsp60 levels in type I fibers in the *gastrocnemius* and the *soleus* of trained mice compared to sedentary mice at 45 days (*P* < 0.01). The mosaic staining pattern and the increase in the levels of Hsp60 in type I fibers in TR45 mice compared to the control, displayed in Fig. 2B, was also demonstrated by confocal microscopy, Fig. 3A. Type I fibers were stained with anti-MHC-I antibody, and by the double fluorescence method the content of Hsp60 for each fiber was determined, using the Leica application suite advanced fluorescences software; excluding the interstitial cells (Fig. S1 A). Fibers histologically classified as type I were strongly positive for the Hsp60 staining, and there was a significant difference between trained and sedentary mice (P < 0.05) (Fig. 3B). In immunofluorescence images it was evident that Hsp60 was present also in the interstitial cells, and that inside the fibers Hsp60 was localized in subsarcolemmal (SS) and inter-myofibrillar (IMF) mitochondria (Fig. 3C,D). IMF mitochondria appeared as Hsp60 positive spots inside myofibrils, while SS mitochondria as Hsp60 positive bundles beneath the plasma membrane.

### Endurance exercise increases the Hsp60 levels in the soleus and in the blood

**Figure 4:**
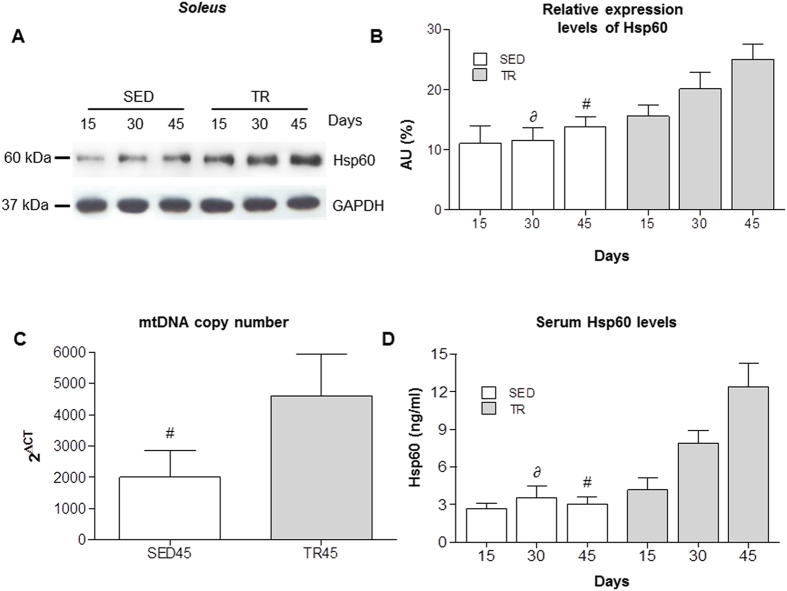
Hsp60 protein levels increase in parallel with training intensity in the soleus muscle and in the blood stream of trained mice. (**A**) representative western blots of Hsp60 (60 kDa) in the *soleus* from the trained (n = 8) and sedentary mice (n = 8) at various time points. 40 μg of protein was loaded in each lane; GAPDH (37 kDa) was used as the loading control. (**B**) relative levels of Hsp60 in the *soleus.* Open bars, sedentary mice; shaded bars, trained mice; horizontal axis, days. AU: Arbitrary Unit. (**C**) copy number of mitochondrial genes in the *soleus* of sedentary mice (n = 6) at day 45 (SED45, open bar) and trained mice (n = 6) at day 45 (TR45, shaded bar). (**D**) serum levels of Hsp60 in SED (n = 8) and TR (n = 8) groups at various time points. Open bars, sedentary (SED) mice; shaded bars, trained (TR) mice; horizontal axis, days. Data are presented as the means ± SD. ∂ significantly different from TR30 mice (P < 0.05). # significantly different from TR45 mice (P < 0.001).

**Figure 5:**
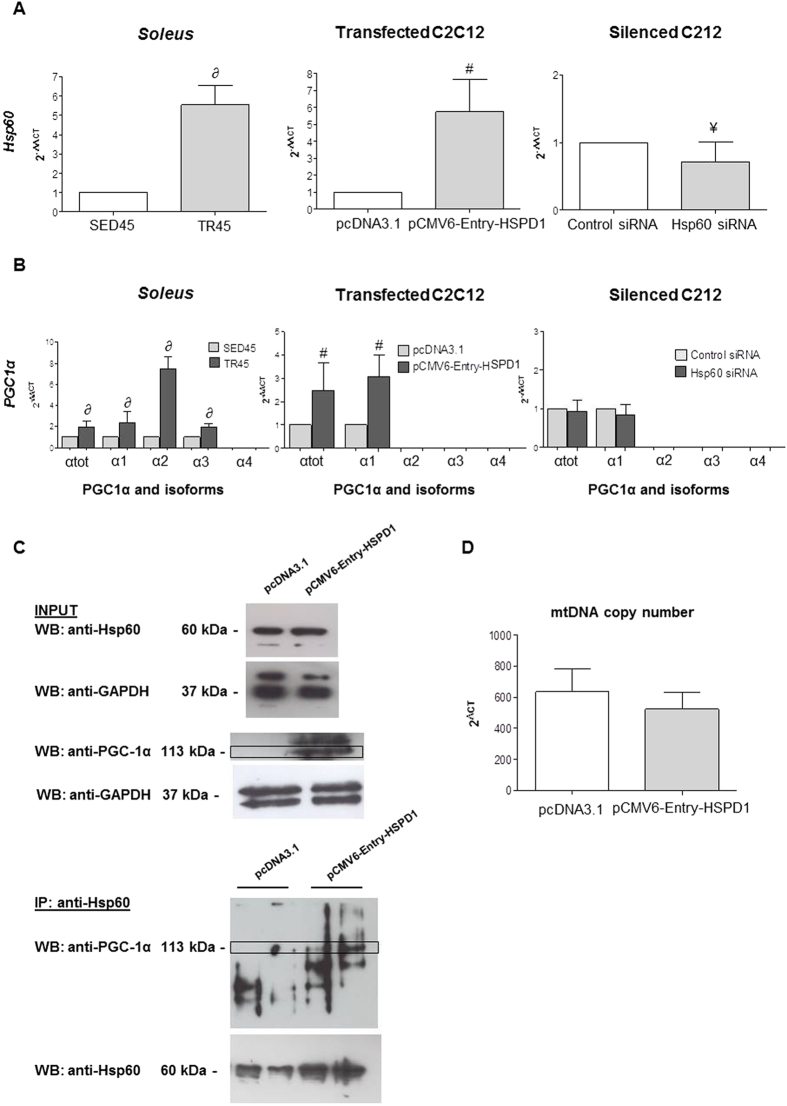
qRT-PCR analysis validates the increase in the levels of *hsp60* gene expression, shows the increase in the levels of *PGC1α* gene expression and its isoforms in the *soleus* of trained mice, and suggests a possible correlation between *hsp60* and *PGC1 α1* genes in HSPD1 transfected C2C12 cells. (**A**) bars show the *hsp60* gene expression levels normalized for the reference genes, according to the Livak Method (2^−∆∆CT^) (Schmittgen and Livak, 2008) in: *soleus* of sedentary (n = 6) and trained (n = 6) mice at 45 days (SED45, and TR45, respectively); C2C12 myoblasts transfected with pCMV6-Entry-HSPD1 vector to over-express *hsp60* (pcDNA3.1 was used as a negative control); and Hsp60 siRNA for silencing Hsp60 (scramble siRNA used as a negative control, Control siRNA). (**B**) bars show the *PGC1α* isoforms [*PGC1α* total (α_tot_), isoform 1 (α_1_), 2 (α_2_), 3 (α_3_), 4 (α_4_)] gene expression normalized for the reference genes, according to the Livak Method (2^−∆∆CT^), in: *soleus* of SED45 (grey bars) and TR45 (black bars); C2C12 myoblast transfected with pCMV6-Entry-HSPD1 vector to over-express *hsp60* (pcDNA3.1 was used as a negative control); and Hsp60 siRNA for silencing Hsp60 (scramble siRNA used as a negative control, Control siRNA). Data are presented as means ± SD, bars show the *PGC1α* isoforms [*PGC1α* total (P < 0.01); ∂ significantly different from SED45 (P < 0.01); ^#^significantly different from pcDNA3.1 (P < 0.01); ^¥^significantly different from Control siRNA (P < 0.01). (**C**) Upper panel: western blot analysis for endogenous expression levels of both Hsp60 and PGC1α in whole-cell lysates from C2C12 myoblasts transfected with pCMV6-Entry-HSPD1 vector to over-express *hsp60* (pcDNA3.1 was used as a negative control). GAPDH was used as a control for loading. Lower panel: representative blot of immunoprecipitation experiments in C2C12 myoblasts transfected with pCMV6-Entry-HSPD1 vector to over-express *hsp60* (pcDNA3.1 was used as a negative control) showing a 113 kDa band corresponding to *PGC1α* that co-immunoprecipited with Hsp60. D, copy number of mitochondrial genes in C2C12 myoblast transfected with pCMV6-Entry-HSPD1 vector (shaded bar) (pcDNA3.1 was used as a negative control, open bar).

The Western blot analysis of the posterior muscle group, described above, revealed no differences in Hsp60 protein levels. Considering that many fibers positive for Hsp60 were detected in the *soleus*, and that this muscle is richer in type I, IIa, and IIx fibers compared to other posterior muscles, the effect of endurance exercise on Hsp60 protein levels were evaluated in the *soleus* (Fig. 4A). This analysis revealed a significant increase in Hsp60 levels in the trained mice compared to the sedentary mice. A significant difference was detected between the TR30 and SED30 mice (*P* < 0.05) and the TR45 and SED45 mice (*P* < 0.001) (Fig. 4B). The increase in Hsp60 was confirmed by quantitative Real-Time PCR (qRT–PCR) (Fig. 5A).

Hsp60 is considered a biomarker of mitochondria. To determine if the increase in the levels of Hsp60 was paralleled by an increase in the number of mitochondria, we evaluated the copy number of mitochondrial genes compared to the nuclear ones. This analysis was performed by qRT-PCR as described in the Material and Methods section. TR45 mice showed more than double of mitochondria compared to SED45 (Fig. 4C). The increases in mitochondrial DNA and in Hsp60 levels in muscle were accompanied by elevated levels of Hsp60 in the blood of trained mice, which showed blood Hsp60 levels higher than sedentary mice at 30 and 45 days (Fig. 4D).

### Effect of endurance training and forced expression of the hsp60 (HSPD1) gene on the levels of PGC1α isoforms

To determine if the increase in Hsp60 protein levels described in the preceding paragraphs was due, at least in part, to a physiological increase in *hsp60* gene expression, we performed qRT-PCR and found that the gene was considerably more expressed in TR45 and in SED45 mice (Fig. 5A). Since the increase in Hsp60 protein and gene expression levels was correlated to an increase in mitochondrial DNA, we decided to evaluate the gene expression and protein levels of the coactivator PGC1α involved in the mitochondria biogenesis pathway. In particular we focused our attention on PGC1α isoforms related to endurance training as recently described^18^. Here, we adopted the same nomenclature used by the authors who described these products for the first time^18^. We called *PGC1 α1* the transcript originating from the first promoter corresponding to a 113 kDa protein; *α2* the transcript originating from the second promoter and corresponding to a 41.9 kDa protein; *α3* the transcript originating from the third promoter and corresponding to a 41.0 kDa protein; and *α4* the transcript originating from the third promoter and corresponding to a 29.1 kDa protein. We performed qRT-PCR, using the same primers reported previously.

In the *soleus* of trained mice the *PGC1α* isoform *α4* was not detected but there was an increase in total *PGC1α*, and in the *α1*, *α2*, and *α3* isoforms (Fig. 5B), which paralleled the increase in *hsp60* gene expression (Fig. 5A).

We found that elevated levels of Hsp60 protein in the *soleus* of trained mice, and increased expression of the *hsp60* gene were accompanied by an increase in the expression of *PGC1α* gene (isoforms *α1*, *α2*, and *α3*). These observations suggested that *hsp60* levels and *PGC1α* gene expression were linked in some way. It was, therefore, pertinent to ask if forced expression of the *hsp60* gene would be accompanied by an increase in the *PGC1α* gene. For this purpose, we used dividing C2C12 myoblasts transfected with pCMV6-Entry-HSPD1 and found a selective increase in the expression of the isoform 1 of *PGC1α* only (Fig. 5B). A close correlation between *hsp60* and *PGC1 α1* gene expression was also suggested by silencing experiments (Fig. 5A,B). The transfection of a siRNA against *hsp60* partially knocked down *hsp60*, and this did not have any effect on the basal levels of PGC1 α1(Fig. 5B).

To understand if the activation of PGC1 α1 was correlated to a possible direct interaction with Hsp60, we immunoprecipitated this protein and performed a Western blotting analysis on the immunoprecipate with the anti-PGC1α antibody. It seemed that at least a 113 kDa PGC1α isoform co-immunoprecipitated with Hsp60 (Fig. 5C).

The increases in the levels of Hsp60 and PGC1 α1 protein and gene expression were not due to an increase in the number of mitochondria inside each cell, as demonstrated by the levels of mtDNA (Fig. 5D).

### Effect of endurance training and forced expression of the *hsp60 (HSPD1)* gene on the protein level of PGC1α, 4 HNE, Mn SOD, p-AMPKα, AMPKα1, AMPKα2 and TFAM

**Figure 6:**
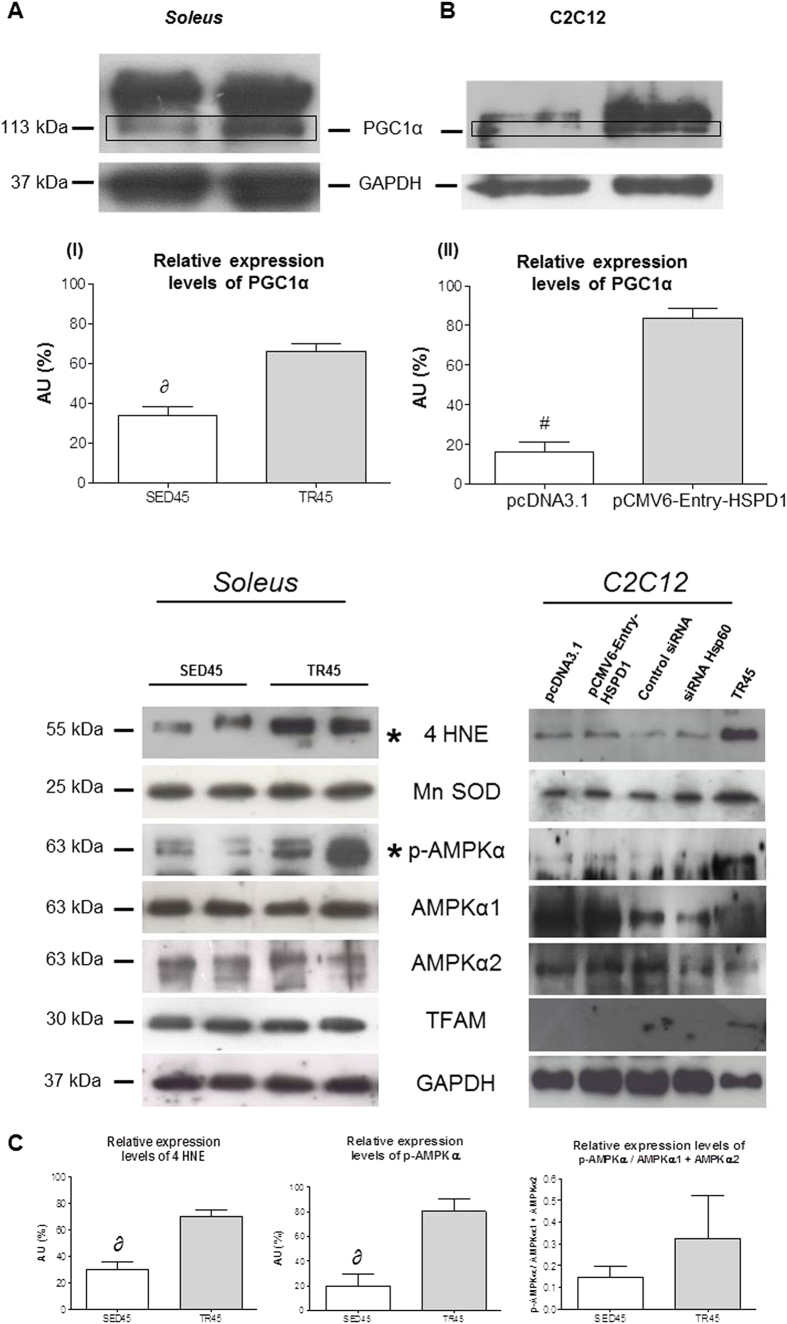
PGC1 α1 levels increase in the *soleus* in trained mice and in transfected C2C12 cells upon transfection with pCMV-Entry-HSPD1 vector. (**A**) representative western blots of *soleus* and relative expression levels (bars) of PGC1 α1 (113 kDa), 4 HNE (55 kDa), Mn SOD (25 kDa), p-AMPKα (63 kDa), AMPKα1 (63 kDa), AMPKα2 (63 kDa), TFAM (30 kDa) in *soleus* of sedentary (SED45, open bar, n = 8) and trained (TR45, shaded bar, n = 8) mice at 45 days. 80 μg of proteins were loaded in each lane; GAPDH (37 kDa) was used as the loading control. Data are presented as the means ± SD. ∂ significantly different from TR45 mice (P < 0.001). AU: Arbitrary Unit. (**B**) representative western blots of C2C12 cells and relative levels (bars) of PGC1 α1 (113 kDa), 4 HNE (55 kDa), Mn SOD (25 kDa), p-AMPKα (63 kDa), AMPKα1 (63 kDa), AMPKα2 (63 kDa), TFAM (30 kDa) in C2C12 myoblast transfected with pCMV6-Entry-HSPD1 vector (pcDNA3.1 was used as a negative control). 80 μg of proteins were loaded in each lane; GAPDH (37 kDa) was used as the loading control and TR45 was used as positive control. Data are presented as the means ± SD. ^#^significantly different from pCMV6-Entry-HSPD1 (P < 0.0001). AU: Arbitrary Unit. *significant results. (**C**) relative expression levels of 4 HNE, p-AMPKα, p-AMPKα/(AMPKα1 + AMPKα2) in the *soleus*. Open bars, sedentary mice; shaded bars, trained mice. AU: Arbitrary Unit.

**Figure 7:**
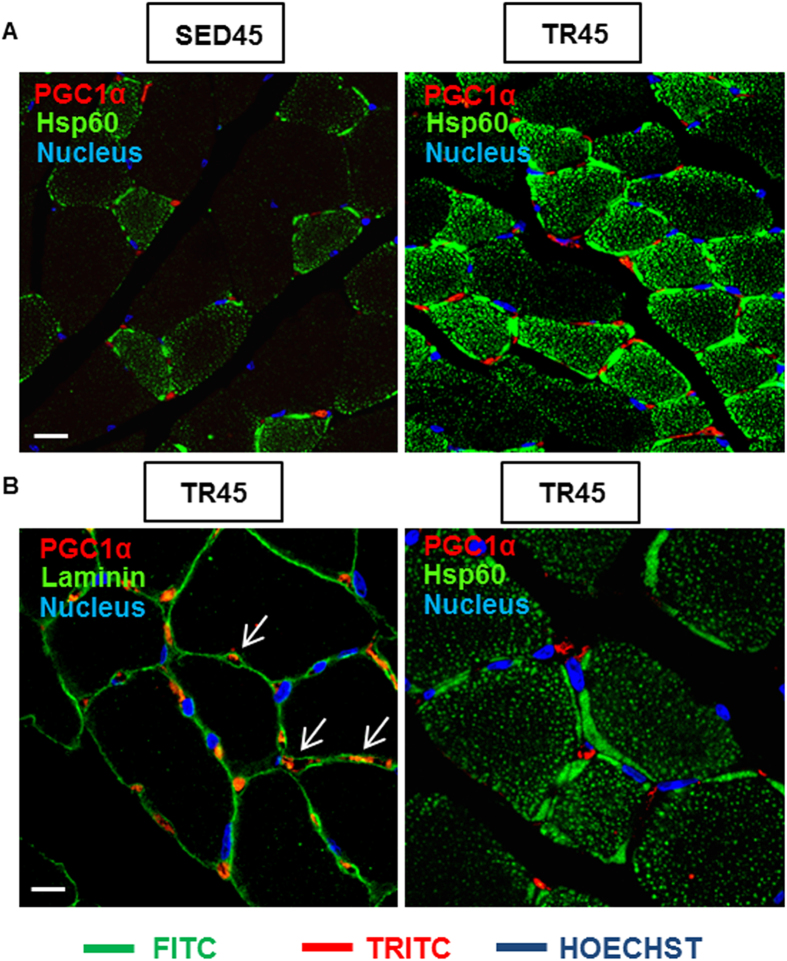
The confocal microscopy analysis shows the localization of PGC1α positive cells in the skeletal muscle tissue. (**A**) immunofluorescence for Hsp60 and PGC1α of muscle cross-sections of sedentary and trained mice at 45 days (SED45 and TR45, respectively). Bar 25 μm. (**B**) immunofluorescence for laminin and PGC1α (the arrows indicate the localization of PGC1α in the interstitial space, outside the fibers; a negative control is shown in supplemental Figure S1B); and Hsp60 and PGC1α of muscle cross-sections of trained mice at 45 days (TR45) at higher magnification. Bar 15 μm.

The increase in PGC1α observed in C2C12 cells transfected with pCMV6-Entry-HSPD1 was similar to that induced by endurance training in the mouse muscle and was confirmed by Western blotting (Fig. 6A) and by confocal microscopy (Fig. 7A).

We also evaluated the protein levels of 4 Hydroxynonenal (4 HNE), manganese superoxide dismutase (Mn SOD), total phospho AMP-activated protein kinase alpha (p-AMPKα), AMPKα1, AMPKα2 and Transcription Factor A Mitochondrial (TFAM) both in the muscle of trained mice and in C2C12 cells transfected with pCMV6-Entry-HSPD1. In the trained muscle increased only 4 HNE and total p-AMPKα, while in transfected C2C12 these proteins did not increase (Fig. 6). Performing an analysis comparing the levels of total p-AMPKα against the sum of the levels of AMPKα1 and AMPKα2, there is not a significant difference between trained and untrained mice (Fig. 6C).

In the skeletal muscle of TR45 mice, PGC1α was localized in the cytoplasm of interstitial cells (mesenchymal progenitors, connective tissue cells or pericytes)^19^. Positive cells resided outside the basal lamina and a few nuclei were positive for PGC1α (Fig. 7B; white arrows). Double immunofluorescence for Hsp60 and PGC1α demonstrated that Hsp60 was at high levels also in interstitial cells and that PGC1α was localized in a cytoplasmic compartment between the nucleus and mitochondria (Fig. 7B). The specificity of the anti-PGC1α antibody was demonstrated by immunofluorescence performed on negative controls, as the one shown in supplemental Figure S1B.

### Exercise induced the release of Hsp60-bearing exosomes in the blood

**Figure 8:**
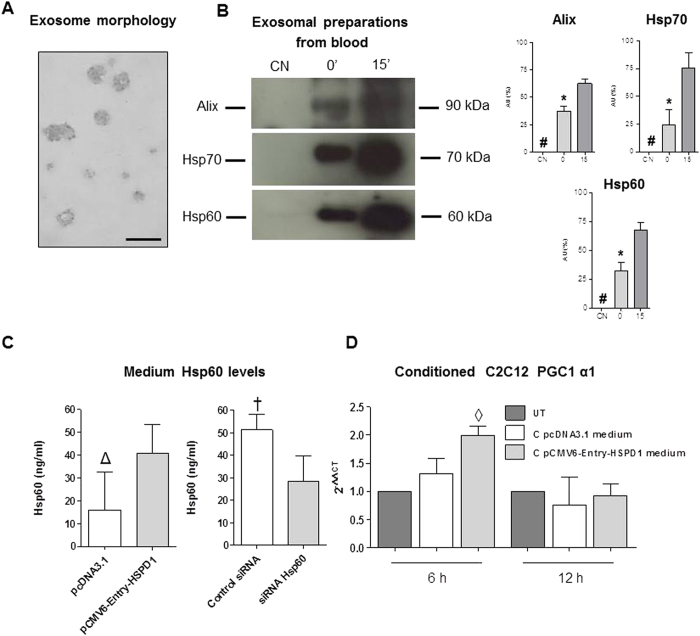
Exosomal preparations from the blood of trained mice and qRT-PCR analysis of *PGC1 α1* gene in C2C12 cells treated with the conditioned media from transfected C2C12 myoblasts. (**A**) Transmission electron microscopy (TEM) image showing the morphology of exosomes purified from blood. (**B**) western blot analysis demonstrating the presence of Hsp60 (60 kDa) and of exosomal markers Alix (90 kDa) and Hsp70 (70 kDa) in the purified exosomes obtained from the blood of twenty-four (four mice for each blood sample) mice submitted to a single bout of exercise and sacrificed immediately (0′) and 15 min (15′) after completing exercise, and sedentary mice (CN). 80 μg of protein was loaded in each lane. Relative expression levels of Alix, Hsp70 and Hsp60 in exosomes derived from blood of trained and sedentary mice. AU: Arbitrary Unit. # significantly different from 0′ and 15′ (P < 0.001). * significantly different from 15′ (P < 0.001).

Since we found increased levels of Hsp60 protein in the blood in trained mice, and since exosomes have been described transporting Hsp60 in circulation^20^, we tested for the presence of Hsp60-carrying exosomes in the blood of mice trained with a single exercise bout and sacrificed immediately or after 15 min. We used blood samples coming from mice after acute exercise and taken within 15 minutes, because release of exosomes is a rapid and quick mechanism which might not be appreciable in trained mice sacrificed 48 hours after the end of the last training session. Anyway to obtain blood for exosomal preparation we needed to train and sacrifice new groups of mice. We found in the exosomal fraction derived from the single bout trained mice the proteins Alix and Hsp70 which are typical exosomal markers. In this exosomal preparation we found also the presence of Hsp60 (Fig. 8A,B). A significant increase was detected in Hsp60, Alix and Hsp70 levels in 15′ and 0′ with respect to CN mice (P < 0.001), and between 0′ and CN mice (P < 0.001) (Fig. 8B).

### Hsp60 released in the culture medium induced the increase in Hsp60 and PGC1α levels in C2C12 cells treated with a conditioned medium

As shown in Fig. 8C, C2C12 cells released Hsp60 in the medium. When transfected with the pCMV6-Entry-HSPD1 plasmid these cells released more Hsp60, while when *hsp60* gene expression was silenced they released less protein.

The conditioned medium was used to treat normal C2C12 cells. Myoblasts after a 6 h treatment showed a significant increase in the levels of expression of the *PGC1 α1* gene.

## Discussion

The data reported here show a differential quantitative distribution of Hsp60 within a single muscle group and changes in this pattern in response to exercise, including its increase in circulation. These data, along with what is known on the diversity of functions of Hsp60 inside and outside cells, provide the tenets for future investigation on the role of the chaperone in muscle physiology and pathology beyond its canonical tasks inside mitochondria.

The major findings in this report are: the quantitative distribution of Hsp60 is distinctive of fiber type; after endurance training Hsp60 increases predominantly in type I fibers and in the blood; endurance training also causes increase in the levels of PGC1α isoforms α1, α2, and α3 but not of the isoform α4; and an increase in the expression of the *hsp60* gene in a cell correlates closely with an increase in *PGC1 α1* gene expression in the same cell.

The fiber-type-specific levels of Hsp60 in skeletal muscle we report is a novel finding. By performing immunohistochemical determination of Hsp60 levels in a cross-section of the entire posterior group of hindlimb muscles, and comparing the content of Hsp60 to that of MHCs, it was possible to unveil differences between muscles and fiber types. Serial cross-sections of the muscles revealed that Hsp60 was elevated in type I, IIa, and IIx muscle fibres, while type IIb fibers appeared only slightly positive for Hsp60. The quantitative distribution pattern of Hsp60 detected by immunohistochemistry was confirmed by means of a quantitative computational analysis that focused on the individual fibers of each muscle of the entire posterior group. Our data contrast with those from another group that reported that type I and type II fibers displayed a similarly uniform cytoplasmic staining pattern^21^. However, these authors indicated that their results were unexpected considering that Hsp60 is a molecular chaperone involved in mitochondrial homeostasis^22^,^23^,^24^ and also considering the differences in mitochondrial content between slow and fast fibers.

The contrast between the lack of a fiber-type-specific quantitative distribution pattern of Hsp60 as reported by others in humans (see above) and our own data showing different levels of Hsp60 in different fibers in mice may be explained by: 1) the quantitative distribution of the fiber types in the human (our work) muscle may differ from that in the mouse; 2) the sample preparation (frozen vs. paraffin embedded in our work); and 3) the antibody specificity (StressGen vs. Abcam in our work).

Based on immunohistochemical and confocal images of the entire posterior group of hindlimb muscles, and western blotting analyses of isolated mouse *soleus* muscle (particularly rich in type I, IIa, and IIx fibres compared to the other muscles studied, the *gastrocnemius* and the *plantaris*), it can be said that our study demonstrates that there is an increase in the levels of Hsp60 in muscles from trained mice as compared with sedentary counterparts, and the level of the chaperone augments with increased training.

The quantitative computational analysis performed on immunohistochemical images showed that Hsp60 increased preferentially in type I fibers in trained mice in all the analyzed muscles, red *gastrocnemius*, *plantaris*, and *soleus*. The increase of Hsp60 in type I fibers was confirmed by confocal analyses of the same muscles and double staining for Hsp60 and MHC-I, and by analyzing the CSA of each single fiber. The confocal images demonstrated that Hsp60 was at high levels also in interstitial cells. The discrepancy between the results of western blotting and qRT-PCR and those of the immunohistochemical analysis might be due to the presence of many interstitial positive cells that were not included in the latter analysis.

Overall, our results agree with findings from other animal studies that revealed that chronic contractile activity^25^,^26^ and periods of treadmill training^27^ can increase the levels of Hsp60 in muscle. In our study Hsp60 significantly increased exclusively in type I fibers of the red *gastrocnemius* and the *soleus.* The effect of endurance exercise on Hsp60 levels has been detected only in studies that examined muscles rich in type I fibers, such as the *plantaris* and the *soleus* from rats^27^,^28^, and the *soleus* from mice (our results). This increase may represent a physiological adaptation in response to exercise since other normal adaptations caused by aerobic training, such as decreased body weight and increased strength and hypertrophy of type I muscle fibers, were also detected.

In our experiments, the increase in Hsp60 levels due to endurance training in the *soleus* was accompanied by an increase in the levels of mitochondrial DNA compared to nuclear DNA, indicating mitochondrial biogenesis, and by release of the Hsp60 protein in the blood (extra-mitochondrial localization of the protein).

Circulating Hsp60 has been observed in various pathological conditions, such as type 2 diabetes^29^, coronary heart disease^30^, carotid arterial stiffness^31^, periodontal disease^32^, idiopathic left ventricular dysfunction^33^, physiological and psychosocial stress^34^, atherosclerosis^35^, recurrent vulvovaginitis^36^, Hashimoto’s thyroiditis^37^, and in normal and aged individuals^38^,^39^, and after a single bout of exercise^40^. How Hsp60 reaches the blood in normal individuals is not known but we have previously demonstrated that, when accumulated in human tumor cells, Hsp60 is found in the plasma-cell membrane and is released via the lipid raft-exosomal pathway^20^,^41^.

Three isoforms of PGC1α were increased, α1, α2, and α3 but not isoform α4. Two different promoters can drive transcription of the *PGC1α* gene in mouse skeletal muscle^42^. A proximal promoter is located upstream of a canonical exon 1 (exon 1a) and a distal promoter followed by an alternative exon 1 (exon 1b) is located 13.7 kb upstream from the exon 1a of the *PGC1* gene, Figure S3. The transcription starting from the proximal promoter induces the expression of *PGC1 α1*^18^, also known as *PGC1 α-a*^43^, and of *NT-PGC1 α-a*^43^; while the transcription starting from the upstream alternative promoter induces the expression of other isoforms: *PGC1 α2, PGC1 α3*, and *PGC1 α4*^18^; and *PGC1 α-b, PGC1 α-c, NT-PGC1 α-b* (known also as *PGC1 α4*), and *NT-PGC1 α-c*^43^. The NT isoforms are the results of an alternative splicing between the exons 6 and 7^43^.

We studied the levels of the PGC1α isoforms transcribed either by the activation of the proximal or the alternative promoter, or as a result of an alternative splicing of exon 1. We found higher levels of the isoform α2 in trained mice (TR45) compared to sedentary counterparts (SED45). These results are in contrast with those of other studies in which higher levels were observed for isoform α1 compared to isoform α2 in resistance training experiments^18^. This discrepancy with our results may be due to a difference in the training protocol used by us, as compared to that applied by the other investigators.

*NT-PGC1α* alternative splicing isoforms (*NT-PGC1 α-a*, *b*, and *c*) mRNAs may be induced by a single bout of high, medium, and low intensity exercise^43^. By using a pan anti-body against all PGC1α isoforms we did not detect any 35 kDa band corresponding to *NT-PGC1α* isoforms, and we never detected *PGC1 α4*.

These results indicate that a prolonged exercise causes exclusively, or predominantly, an increase in the levels *PGC1 α1*, *α2*, and, *α3* isoforms, with the greatest increase being that of the *α2* isoform.

Confocal analysis demonstrated that PGC1α is localized to the cytoplasm of interstitial cells, in an area between mitochondria and the nuclear membrane. We did not characterize interstitial cells apart for the presence of their basal laminae. No nuclei of the interstitial cells were positive for PGC1α, and this was also the case for satellite cells. We did not find any publication showing by immunofluorescence or immunohistochemistry a nuclear localization of PGC1α in the fibers of the skeletal muscle tissue, although nuclear localization of PGC1α did appear in cultured cells after 1 h treatment with hydrogen peroxide (H_2_O_2_)^44^. We detected PGC1α in the cytoplasm of interstitial cells in the skeletal muscle tissue by immunofluorescence on cryostat sections (image not shown), while in C2C12 cells treated with 200 μM H_2_O_2_ PGC1α translocated to the nucleus after 1 h treatment (Figure S2 A).

A recent paper suggested that cytosolic pools of PGC1α are localized in a defined order inside cells, and that PGC1α may be concentrated in: a) close proximity to the nucleus to be more readily available for translocation upon exercise to the SS mitochondrial fraction, i.e., that which is located in close proximity to the nucleus^45^; or b) in the nucleus itself^46^.

In our experiments, we saw a steady increase in the levels of PGC1α in trained mice and the close-to-nucleus localization. However, we did not see nuclear or mitochondrial localization of the protein most likely because we sacrificed the animals 48 h after the last training session instead of 30 min or 3 h as the other research groups did^45^,^46^.

To determine if Hsp60 does have an effect on the expression of *PGC1 α1* we transfected undifferentiated C2C12 skeletal myoblasts with a plasmid constitutively over-expressing the *hsp60* gene. The transfected C2C12 cells showed high expression of *hsp60* and expression of *PGC1* isoform *α1* but no detectable expression of the other three isoforms. This is the first study to establish that forced overexpression of *hsp60* results in the expression of *PGC1 α1*. Moreover, the immunprecipitation of the Hsp60 protein demonstrated an interaction at least with the 113 kDa isoform of PGC1 α. A direct interaction between the two proteins Hsp60 and PGC1 α1, or a co-activating activity of Hsp60 on the promoter of the *PGC1 α1* gene has not yet been described in the literature.

We also performed a Western blotting analysis to determine the levels of AMPKα1, AMPKα2, p-AMPKα, 4 HNE, MnSOD, and TFAM in both skeletal muscle tissue (soleus) and HSPD1-transfected C2C12 cells. AMPK is the kinase which phosphorylates the co-factor PGC1α, which after phosphorylation translocates in the nucleus and binds to TFAM to activate the transcription of target genes, as Mn SOD, and itself^13^. 4 HNE is directly correlated to the increase in reactive species of oxygen (ROS)^47^. As shown in Fig. 6, we assessed an increase in p-AMPKα and 4 HNE in the skeletal muscle, which demonstrated the release of ROS and consequent activation of p-AMPKα, as reported in literature when the mitochondrial biogenesis pathway is activated^13^. Our results support the hypothesis that in transfected C2C12 cells, PGC1 α1 expression is activated but not the mitochondrial biogenesis pathway as demonstrated by the mitochondrial DNA determination. This limited activation suggests that there is a direct correlation between Hsp60 and PGC1 α1.

In our *in vivo* experiments, Hsp60 was found elevated in muscle fibers, while PGC1α was elevated in interstitial cells, suggesting that Hsp60 is released by the muscle fibers into the intercellular space and, thereby, activates PGC1α in the interstitial cells. In our *in vitro* experiments, C2C12 cells with over-expressed Hsp60 simultaneously expressed considerable levels of PGC1 α1. Although this effect of Hsp60 on *PGC1 α1* gene expression coincided in the same cell, our results demonstrate that Hsp60 is released into the culture medium by a cell and, acting as a paracrine factor, activates PGC1 α1 synthesis in nearby cells. It is likely that the release of Hsp60 by the skeletal muscle tissue, or by C2C12 cells, occurs via exosomes. We have previously demonstrated that Hsp60 can be released in the interstitial space inside exosomes by H292 human tumor cells in culture^20^, and, on the other hand, that C2C12 myoblasts are able to release exosomes in the culture medium^48^. Thus, we can hypothesize that exercise induces the release of Hsp60-bearing exosomes into the bloodstream.

Further experiments are needed to understand if the differentiated muscle is able to release exosomes in the bloodstream and the fate of these Hsp60-bearing exosomes.

## Materials and Methods

### Animals and animal care

One hundred and two young (7-weeks old) healthy male mice (BALB/c AnNHsd), obtained from Harlan Laboratories, Srl (Udine, Italy), were maintained in a 12-hours light-dark cycle and were allowed free access to food and water. After 1 week of acclimatization to the new housing environment, the mice were assigned to one of the two experimental groups: SED or TR. The TR mice were either trained according to a running endurance training protocol for rodents previously performed in our lab^49^ or submitted to a single bout of endurance exercise, while SED mice were not submitted to any of the above. The mice of both groups were free to move inside the cages, which did not contain a rodent running wheel. All animal experiments were approved by the Committee on the Ethics of Animal Experiments of the University of Palermo and adhered to the recommendations in the Guide for the Care and Use of Laboratory Animals by the USA National Institute of Health (NIH). All experiments were performed in the Human Physiology Laboratory of the Department of Experimental Biomedicine and Clinical Neurosciences of the University of Palermo, which was formally authorized by the Italian Ministry of Health (Roma, Italy).

### Endurance training

A motorized Rota-Rod (Rota-Rod; Ugo Basile, Biological Research Apparatus, Comerio Varese, Italy) was used to train the mice^50^. The Rota-Rod is a rotating cylinder on which the mice are forced to run to avoid falling down^51^. The TR mice ran 5 days/week for 6 weeks at a progressively increasing duration and intensity of training: the first week the mice ran for 15 min at a speed of 3.2 m/min; the second week, the mice ran for 30 min at the same speed. The training duration and intensity were systematically increased until week 6, when the mice ran at a speed of 4.8 m/min for 60 min. Every two weeks, all mice were weighed, and their strength was measured. SED15, SED30, TR15 and TR30 (eight mice/group) were sacrificed after 15, 30 days. SED45 and TR45 (twenty-three mice/group) were sacrificed after 45 days. Forty-eight hours after the last training session, the mice were sacrificed via cervical dislocation, and the group of posterior muscles (*gastrocnemius*, *soleus*, and *plantaris*) of the hindlimbs were dissected and preserved in liquid nitrogen (right hindlimb) or embedded in paraffin (left hindlimb) to evaluate the morphological and molecular changes.

### Single bout of endurance exercise

Twenty-four mice ran on a Rota-Rod for 60 min at a speed of 5.8 m/min. Mice were sacrificed by cervical dislocation immediately and 15 min after the single bout of endurance exercise. The blood was collected in EDTA (ethylenediaminetetraacetic acid)-treated tubes and centrifuged at 2,000xg for 10 min at 23 °C^37^, and plasma was stored −80 °C. The plasma was used to isolate and characterize exosomes.

### Grip strength test

The strength of the forelimbs was measured using a grip strength meter (47200-Grip-Strength Meter, Ugo Basile) every two weeks. Each mouse was tested five times in succession and the best three results were averaged, following the grip test protocol as previously described^52^.

### Cell culture methods

The mouse myoblast cell line C2C12 was maintained in high glucose DMEM supplemented with 20% fetal bovine serum (FBS), sodium pyruvate, L-glutamine, penicillin, and streptomycin (proliferation medium) at 37 °C in a humidified atmosphere with 5% CO_2_. Plasmids were transfected using Lipofectamine® 2000 Reagent according to the manufacturer’s instructions. Briefly, C2C12 cells were plated 1 day before transfection in 6-well dishes (5 × 10^4^ cell/well). On the day of the transfection, the cells were 70% confluent. Three point five (3.5) μg plasmid DNA [pCMV6-Entry-HSPD1] for overexpression of Hsp60 (OriGene Technologies Inc., Rockville, MD, USA) and pcDNA 3.1 plasmid for its negative control was diluted in 150 μl of Opti-MEM Medium (without FBS, L-glutamine, penicillin, or streptomycin) and mixed with 6 μl of Lipofectamine diluted in 150 μl of Opti-MEM. The mixture was added to growing cells. After 7–8 h, Opti-MEM and FBS was added to a final concentration of 20% in a final volume of 2 ml. The transfection is shown in Figure S2 B.

Mouse Hsp60 siRNA (sc-35604, Santa Cruz Biotechnology, Inc. Dallas, Texas, USA) was administered according to siRNA Reagent System protocol (sc-45064, Santa Cruz Biotechnology), using a mixture of 8 μl siRNA duplex diluted in 100 μl Transfection Medium, with 8 μl siRNA Transfection Reagent diluted in 100 μl Transfection Medium. The siRNA negative control was used in the same way. After 7 hours, 1 ml of normal growth medium was added. The cells were incubated for 48 h.

To extract proteins and RNA, cells from each well were re-plated into a new 6-well plate and incubated for another 24 h in proliferation medium. Moreover, naïve C2C12 cells were incubated with the conditioned medium from cells overexpressing Hsp60 for 6 and 12 h to extract RNA.

For immunofluorescence analysis, 10^4^ cells/well were plated in chamber slides and after 24 h cells were fixed with 4% glutaraldehyde and icy methanol.

### Immunoblotting

Skeletal muscle homogenization was performed with frozen sections either of the posterior muscle group of the hindlimbs (approximately 250 mg each, containing *gastrocnemius*, *soleus*, and *plantaris* muscles) or of the *soleus* only. Muscles were homogenized by hand (mortar and pestle) in an ice-bath in lysis buffer (200 mM HEPES, 5 M NaCl, 10% Triton X-100, 0.5 M EDTA, 1 M DTT, 0.25 g Na-deoxycholate, 0.05 g SDS) supplemented with Protease Inhibitor Cocktail (Sigma-Aldrich, St. Louis, MO, USA). C2C12 cells were homogenized in the same lysis buffer of skeletal muscle supplemented with Protease Inhibitor Cocktail and both the homogenates were centrifuged at 13,000xg for 15 minutes at 4 °C and the supernatant fractions (total lysate) were stored at −80 °C. The protein concentrations were quantified spectrophotometrically according to Bradford (Bradford, 1976) using bovine serum albumin (BSA, Sigma-Aldrich) as the standard.

The proteins were separated in 12% SDS-PAGE and electrophoretically transferred to a nitrocellulose membrane 0.45 μm (Bio-Rad Laboratories, Segrate Milano, Italy). The membrane was incubated in a blocking solution containing 5% BSA in Tris-buffered saline (20 mM Tris, 137 mM NaCl, pH 7.6) containing 0.05% Tween-20 (T-TBS) for 1 h at 23 °C. Next, the membrane was further incubated in a primary antibody, anti-Hsp60 (diluted 1:1,000, mouse monoclonal antibody ab13532, Abcam, Cambridge, UK), or anti-glyceraldehyde-3-phosphate dehydrogenase (GAPDH) diluted 1:3,000, rabbit polyclonal antibody ADI905784, Enzo Life Sciences, Inc. NY, USA), or anti-PGC1α (diluted, 1:1,000, mouse monoclonal antibody ST1202, Calbiochem, Inc. San Diego, CA, USA), or anti-Hsp70 (diluted, 1:1,000, mouse monoclonal antibody C92F3A-5, Santa Cruz Biotechnologies), or anti-Alix (diluted, 1:500, mouse monoclonal antibody sc-53538, Santa Cruz Biotechnologies), or anti-Mn SOD (diluted 1:1000, rabbit polyclonal antibody ADI-SOD-110, Enzo Life Sciences), or anti- AMPKα1 (diluted 1:1000, rabbit polyclonal antibody 07-350SP, Millipore, Temecula, CA, USA), or anti-AMPKα2 (diluted, 1:1000, rabbit polyclonal antibody 07-363SP, Millipore), or anti-phospho-AMPKα (diluted, 1:1000, rabbit polyclonal antibody 07-681SP, Millipore), or anti-TFAM (diluted 1:1000, rabbit polyclonal antibody DR1071, Calbiochem, Inc. San Diego, CA, USA), or anti-4 HNE (diluted 1:1000, rabbit polyclonal antibody, ab46545, Abcam). All the primary antibodies were diluted in T-TBS containing 0.5% BSA and incubated overnight at 4 °C. The following day, the membrane was washed with T-TBS and incubated with an HRP-conjugated secondary antibody (anti-rabbit NA934V, or anti-mouse NA931, Amersham Biosciences, NY, USA; for Fig. 6B anti-rabbit A-6154, Sigma ImmunoChemicals, MO, USA) diluted in T-TBS containing 0.5% BSA for 1 h. The detection of the immunopositive bands was performed using ECL Western Blotting Detection Reagent (Amersham Biosciences) according to the manufacturer’s instructions. The detected bands were analyzed using ImageJ software version 1.41 (NIH, USA; http://rsb.info.nih.gov/ij). GAPDH bands were checked to control loading. Full blots are shown in Figure S4.

### Immunoprecipitation

In order to detect the interaction between Hsp60 and PGC1α in C2C12 cells transfected with pCMV6-Entry-HSPD1, immunoprecipitation was performed. Briefly, equal amounts of protein (500 μg) from total cells lysates were incubated with the primary antibody (rabbit polyclonal anti-HSP60, H-300, sc-13966, Santa Cruz Biotechnology) overnight at 4 °C with gentle rotation. Antibody/protein complexes were then immunoprecipitated with antibodies linked to Sepharose A beads (GE Healthcare, Milano, Italy). Nonspecifically bound proteins were removed by repeated washings with isotonic lysis buffer. Immunoprecipitated proteins were resolved by 10% SDS-PAGE using anti-PGC1α primary antibody (diluted, 1:1,000, mouse monoclonal antibody ST1202, Calbiochem).

### Immunohistochemistry

Muscles were fixed in a solution of acetone, methanol, and water (2:2:1) for 12 h, washed in tap water and dehydrated with ethanol at 70, 96, 100% v/v. After dehydration, the tissue pieces were placed in xylol for 1.5 h and embedded into paraffin. The embedded muscles were sliced into sections (5 μm) that were mounted on glass slides. For immunohistochemical analysis, the serial sections were incubated in an “antigen unmasking solution” (10 mM tri-sodium citrate, 0.05% Tween-20) for 10 min at 75 °C. Then, the MACH1 kit (M1u539g, Biocare, Concord, CA, USA) was used according to the manufacturer’s instructions. The sections were incubated in primary antibodies, including anti-myosin heavy chain-I (MHC-I, A4951, Hybridoma Bank, Iowa, IA, USA), anti-myosin heavy chain-II (MHC-II, A474, Hybridoma Bank), and anti-Hsp60 (rabbit polyclonal antibody ab53109, Abcam), in a humidified chamber overnight at 4 °C. The following day, the sections were incubated for 1 h with the secondary antibody and polymers as previously described^53^. Finally, the slides were coverslipped, and images were captured with a Leica DM5000 upright microscope (Leica Microsystems, Heidelberg, Germany). The CSA of the type I muscle fibers of the posterior muscle group from the hindlimbs was measured using ImageJ 1.41 software to evaluate the effect of endurance training. The analyses were performed using 5 fields per section, 5 sections per mice (40 μm between sections) and 8 mice per group. An average of 435 fibers was analyzed for each mouse. Densitometric analysis of the staining intensity by the anti-Hsp60 antibody was performed using ImageJ 1.41 software. The acquired image (RGB) was transformed to greyscale (32-bit) and then inverted (Fig. 2C). In the 0-255 greyscale image, 0 corresponds to no positivity and 255 corresponds to maximum positivity; the staining intensity of each measured fiber was expressed as the pixel intensity (PI) normalized to that of the CSA.

### Hsp60 Enzyme-linked Immunoadsorbent Assay (ELISA)

Polyvinylchloride microtiter plates (High bond cod. 353279, BD Biosciences San Jose, CA, USA) were coated with H-300, rabbit polyclonal antibody raised against amino acids 274-573 mapping at the C-terminus of Hsp60 of human origin [H-300, sc-13966, Santa Cruz Biotechnology, 10 μg/ml in PBS (phosphate buffered saline), 50 μl/well, overnight at 4 °C]. All subsequent steps were carried out at 23 °C. After washing three times with PBS, the plates were blocked with 3% BSA in PBS (200 μl/well, 2 h at 23 °C) and washed with PBS again. Hsp60 standard, serum (diluted 1:25; 1:12.5; 1:6.25; 1:3.12) and C2C12 cell culture media in PBS containing 0.5% BSA, 0.05% (v/v) Tween 20 (“assay buffer”) were added (100 μl/well) and incubated for 1.5 h at 37 °C. The plates were washed eight times by emptying and filling with PBS containing 0.05% (v/v) Tween 20 and incubated with goat polyclonal affinity purified antibody raised against a peptide mapping near the C-terminus of Hsp60 of mouse origin (k19, sc-1722, Santa Cruz Biotechnology), 1:1,000 in assay buffer (50 μl/well, 1.5 h at 23 °C). The plates were washed again as above and further incubated with rabbit anti-goat-peroxidase conjugate antibody (1:3,000 in assay buffer, 50 μl/well, 1 h at 23 °C, A-8919, Sigma-Aldrich). After the final wash, the plates were incubated with 0.4 mg/ml o-phenylenediamine (P-8287, Sigma-Aldrich) solution in 0.05 M phosphate-citrate buffer, pH 5.0, containing 3.5 mM hydrogen peroxide (100 ml/well, 30 min). The reaction was stopped by adding 10% (v/v) sulfuric acid (100 μl/well), and the absorbance of each well was read at 492 nm. Each assay was calibrated with eight Hsp60 solutions at various concentrations. Serum and cell media concentrations (in ng/ml) of Hsp60 were calculated according to the standard curves obtained by using recombinant human Hsp60 (V13-31176, Vinci-Biochem srl, Firenze, Italy) in the sandwich ELISA.

### Immunofluorescence and confocal analysis

For immunofluorescence, deparaffinized sections and fixed cells were incubated in the “antigen unmasking solution” (10 mM tri-sodium citrate, 0.05% Tween-20) for 10 min at 75 °C or 23 °C, respectively, and treated with a blocking solution (3% BSA in PBS) for 30 min. Next, the primary antibody (anti-Hsp60, rabbit polyclonal ab53109, Abcam; anti-MHC-I, mouse monoclonal A4.951, Hybridoma Bank; anti-PGC1 alpha, mouse monoclonal ST1202, Calbiochem; anti-laminin, rabbit polyclonal AB2034, Millipore; anti-DDK synthetic peptide (DYKDDDDK), mouse monoclonal TA5001, OriGene) diluted 1:50, was applied, and the sections were incubated in a humidified chamber overnight at 4 °C. Then, the sections were incubated for 1 h at 23 °C with a conjugated secondary antibody (anti-rabbit IgG–FITC antibody produced in goat, F0382, Sigma-Aldrich; anti-mouse IgG-TRITC antibody produced in goat, T5393, Sigma-Aldrich). Nuclei were stained with Hoescht Stain Solution (1:1,000, Hoechst 33258, Sigma-Aldrich). The slides were treated with PermaFluor Mountant (Thermo Fisher Scientific, Inc. Waltham, MA, USA) and cover slipped. The images were captured using a Leica Confocal Microscope TCS SP8 (Leica Microsystems).

### Total RNA and DNA isolation

The total RNA and total DNA (genomic and mtDNA) were extracted using the Tri-reagent (Sigma-Aldrich) according to the manufacturer’s instructions, from cryopreserved samples of transfected cells, conditioned medium treated cells and muscles of trained and sedentary mice. RNA and DNA concentrations in the samples were determined spectrophotometrically.

### Quantitative real-time PCR

Reverse transcription was performed using the ImProm-II Reverse Transcriptase Kit (Promega, Madison, WI, USA) according to the manufacturer’s instructions. qRT–PCR analysis was performed using GoTaq qPCR Master Mix (A6001, Promega). mRNA levels were normalized to that of GAPDH. Changes in the transcript level were calculated using the 2^−ΔΔCT^ method^54^. Complementary deoxyribonucleic acid (cDNA) was amplified using primers indicated in Table S1 (see Supplementary information). cDNA was amplified using the Rotor-gene™ 6000 Real-Time PCR Machine (Qiagen GmbH, Hilden, Germany).

PCR fragments purified using the Nucleospin PCR and Gel Clean-up Kit (Macherey-Nagel GmbH & Co. KG, Düren, Germany) were sequenced by Eurofins Sequencing Service (Edersberg, Germany). Sequences were then analyzed with the BLASTn Web Tool on the NIH website (http://www.ncbi.nlm.nih.gov/BLAST/). All PCR-amplified fragments corresponded to the desired target.

### Relative copy number method

This method compares the levels of nuclear to mitochondrial DNA (mtDNA) in a DNA sample. Real-time PCR provides a platform for a quantitative assay measuring and comparing mtDNA copy number to that of nuclear DNA (nDNA). The amplification primers have been validated for matching amplification efficiency and absence of amplification of mitochondrial pseudogenes by sequencing PCR products and comparing amplicon sequences with BLASTn database. Three optimized PCR primer pairs targeting two mitochondrial (mt-12S and mt-Cyt_b) and one nuclear (BECN1) gene were used. Resultant Cts obtained from the Real-Time instrument are used to represent the level of each gene. Nuclear gene is compared once to that of mitochondrial mt-12S and once to mitochondrial mt-Cyt_b. In this way, ratios are calculated which can be averaged to represent the mtDNA copy number per cell.

Purity of DNA samples was verified by measuring the 260 nm/280 nm ratio with a UV/vis Spectrophotometer. 2 ng of DNA was used in each qRT-PCR reaction tube. qRT–PCR analysis was performed using GoTaq qPCR Master Mix (A6001, Promega).

Cts obtained for each of the three targets were used to determine mtDNA copy numbers. This was accomplished by averaging the copy numbers calculated from mt-12S/BECN1 pair and the mt-Cyp_b/BECN1 pair. The Cts from the mt-12S gene were subtracted from the BECN1 Ct to obtain ΔCt_1_. Likewise, mt-Cyt_b was subtracted from BECN1 to obtain ΔCt_2_. Copy numbers were calculated based on the ΔCt of the matched mitochondrial to nuclear DNA Cts. To calculate copy number, the average of Set 1 = (mt-12S/BECN1) and Set 2 = (mt-Cyt_b/BECN1) ratios were determined. The individual ratios from Set 1 and Set 2 were used to calculate N = 2^ΔCt^ where ΔCt_1_ = Ct^Nucl^-Ct^Mito1^ and ΔCt_2_ = Ct^Nucl^-Ct^Mito2^. Primers used are listed in Table S1.

### Isolation and validation of exosomes from the blood

To obtain a sample for exosomes’ analysis, lipid vesicles were isolated from the plasma of four mice for each sample according to a published method^55^. Twenty-four animals were used for these analyses. Briefly, after centrifugation of plasma at 300 g for 10 min, supernatants were centrifuged at 1,200 g for 20 min followed by 10,000 g for 30 min. Supernatants were filtered using a 0.22 μm filter (Millipore) and centrifuged at 100,000xg for 1 h in a Sorvall™ WX + Ultracentrifuge Series (Thermo Scientific) in order to pellet exosomes. After one wash in a large volume of PBS, exosomes were resuspended in PBS (50–100 μl) or in lysis buffer for immunoblotting, and stored at −80 °C until use. Proteins in exosomal preparations were quantified with the Quant-iT™ protein assay kit (Invitrogen Molecular Probes, Milano, Italy), using the Qubit fluorometer according to the manufacturer’s instructions (the kit is accurate for protein concentrations ranging from 12.5 μg/ml to 5 mg/ml).

Exosome pellets purified were examined by transmission electron microscopy (TEM) according to a previously published method (Merendino *et al.* 2010) to ascertain the presence of exosomal vesicles. Pellets were resuspended in residual fluid from a PBS wash, followed by addition of 100 ml ice-cold, freshly made fixative (2.5% glutaraldeyde in PBS) to preserve vesicle structure and morphology. Preparations were mounted on formvar nickel 300-mesh grids by layering grids over 10-ml drops of exosome preparations for 10 min at 23 °C. Grid-mounted preparations were stained with uranyl acetate and lead citrate, and subsequently examined with a Jeol (JEM 1220) TEM at 120 kV. Also, the presence of exosomes was confirmed by measuring the two proteins Alix and Hsp70, which are also considered a marker of^20^,^41^.

Protein from exosomal preparations was separated via immunoblotting, as described above (see Immunoblotting).

### Statistical analyses

Body weight and force data were statistically analyzed via two-way ANOVA for repeated measurements, while all the other data were analyzed via one-way ANOVA for single measurements or t-test. If a significant difference was detected by ANOVA analyses, the data were further evaluated by the Bonferroni post-hoc test.

All statistical analyses were performed using GraphPad Prism^TM^ 4.0 software (GraphPad Software Inc., San Diego, CA, USA). All data are presented as the means ± SD, and the threshold of statistical significance was set at p < 0.05.

## Additional Information

**How to cite this article**: Barone, R. *et al.* Skeletal muscle Heat shock protein 60 increases after endurance training and induces *peroxisome proliferator-activated receptor gamma coactivator 1 α1* expression. *Sci. Rep.* **6**, 19781; doi: 10.1038/srep19781 (2016).

## Supplementary Material

Supplementary Information
