# Supplementary material for: Skeletal muscle Heat shock protein 60 increases after endurance training and induces peroxisome proliferator-activated receptor gamma coactivator 1 α1 expression
Source: Sci Rep. 2016 Jan 27;6:19781. doi: 10.1038/srep19781 (PMC4728392; doi:10.1038/srep19781)
Supplement: Supplementary Information [file srep19781-s1.pdf]

Supplementary informations

<u>Manuscript</u>

Skeletal muscle Heat shock protein 60 increases after endurance training and induces *peroxisome proliferator-activated receptor gamma coactivator 1 α1* expression

Rosario Barone[1,2], Filippo Macaluso[1,2], Claudia Sangiorgi[1,2], Claudia Campanella[1,2], Antonella Marino Gammazza[1,2], Viviana Moresi[3], Dario Coletti[3,4,5], Everly Conway de Macario[6], Alberto JL Macario[2,6], Francesco Cappello[1,2], Sergio Adamo[3,4], Felicia Farina[1], Giovanni Zummo[1], and Valentina Di Felice[1,2*].

AFFILIATIONS
1 Department of Experimental Biomedicine and Clinical Neurosciences (BioNeC), University of Palermo, Palermo, 90127 Italy; **2** Euro-Mediterranean Institute of Science and Technology (IEMEST), Palermo, 90100, Italy; **3** Department of Anatomical, Histological, Forensic & Orthopaedic Sciences, Section of Histology & Medical Embryology, Sapienza University of Rome, Rome, 00161, Italy; **4** Interuniversity Institute of Myology, Rome, 00161, Italy; **5** University Pierre et Marie Curie Paris, UR4 Aging, Stress, Inflammation, 75005 Paris, France; **6** Department of Microbiology and Immunology, School of Medicine, University of Maryland at Baltimore, IMET, 21201 Baltimore, MD, USA

*vdfelice@inwind.it

Cell cultures

C2C12 cells were cultured in Dulbecco's modified Eagle's medium with 10% FBS and antibiotics. Transfections were performed with Lipofectamine (Invitrogen, Carlsbad, CA, USA) according to the manufacturer's instructions using pcDNA3.1 and pCMV6-Entry-HSPD1 (OriGene). Expression of Hsp60 and DDK was confirmed by confocal analysis (Figure S2B). Knock down of Hsp60 was performed by RNA interference using Silencer pre-designed siRNAs (sc-35604, Santa Cruz Biotechnology), and introduced into cells with Lipofectamine as described (Bongiovanni et al., 2012). To visualize PGC1α nuclear localization, subconfluent cells were exposed to hydrogen peroxide (200 μM) in serum-free media for 1 h. PGC1 alpha localization was studied by confocal microscopy (Figure S2A).

Confocal microscopy

For immunofluorescence, deparaffinized sections and fixed cells were incubated in the "antigen unmasking solution" (10 mM tri-sodium citrate, 0.05% Tween-20) for 10 min at 75 °C or 23 °C, respectively, and treated with a blocking solution (3% BSA in PBS) for 30 min. Next, the primary antibody (anti-Hsp60, rabbit polyclonal ab53109, Abcam; anti-MHC-I, mouse monoclonal A4.951, Hybridoma Bank; anti-DDK, mouse monoclonal TA5001, OriGene; anti-PGC1α, mouse monoclonal ST1202, Calbiochem) diluted 1:50, was applied, and the sections were incubated in a humidified chamber overnight at 4 °C. Then, the sections were incubated for 1 h at 23 °C with a conjugated secondary antibody (anti-rabbit IgG–FITC antibody produced in goat, F0382, Sigma-Aldrich; anti-mouse IgG-TRITC antibody produced in goat, T5393, Sigma-Aldrich). Nuclei were stained with Hoescht Stain Solution (1:1,000, Hoechst 33258, Sigma-Aldrich). The slides were treated with PermaFluor Mountant (Thermo Fisher Scientific Inc.) and coverslipped. The images were captured with a Leica Confocal Microscope TCS SP8 (Leica Microsystems).

References

Bongiovanni A., D.P. Romancino, Y. Campos, G. Paterniti, X. Qiu, S. Moshiach, V. Di Felice, N. Vergani, D. Ustek, and A. d'Azzo. 2012. Alix protein is substrate of Ozz-E3 ligase and modulates actin remodeling in skeletal muscle. J Biol Chem. 287(15):12159-71.

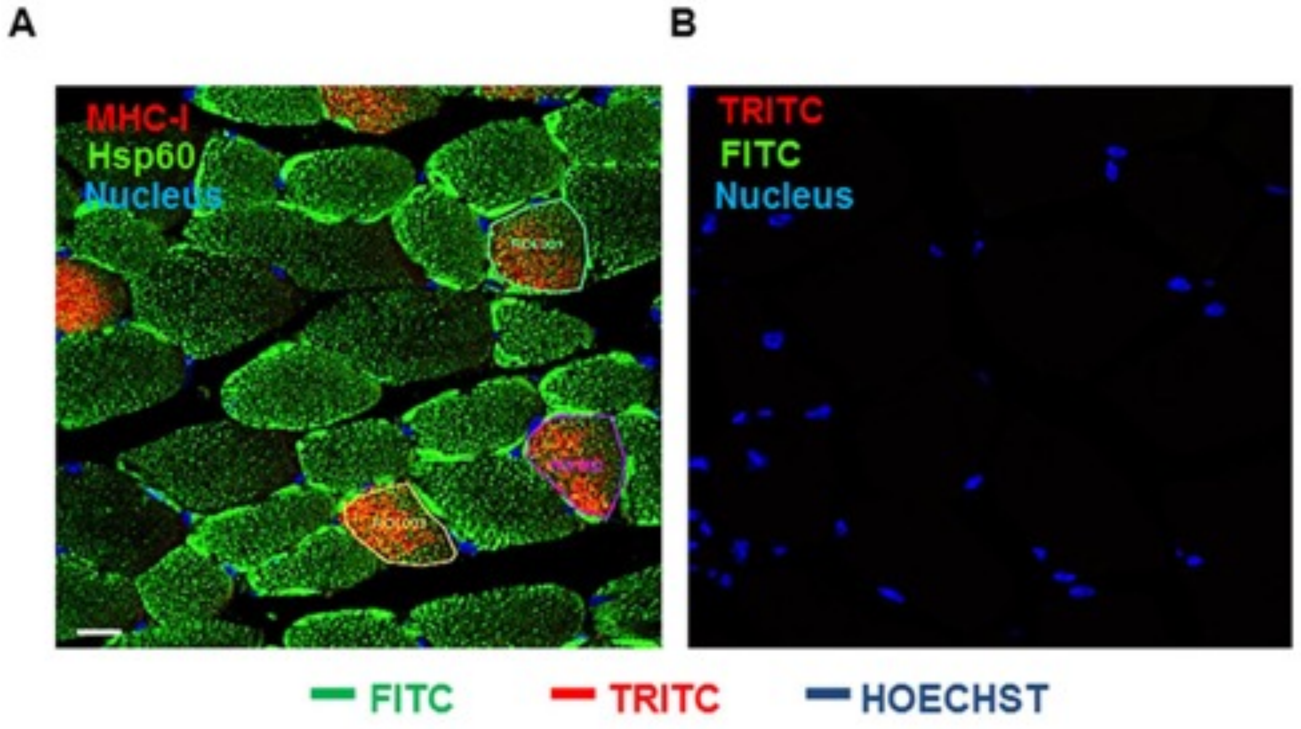

Figure S1. A, An immunofluorescence image for Hsp60 and MHC-I showing how we performed the analysis of the levels of Hsp60 protein in each fiber type by confocal microscopy (in this analysis we excluded the interstitial cells)**.** Bar 25 μm. B, Negative control with an anti-mouse secondary antibody conjugated with TRITC and an anti-rabbit secondary antibody conjugated with FITC showing that PGC1 α staining was not the result of cross-reactions with mouse blood cells.

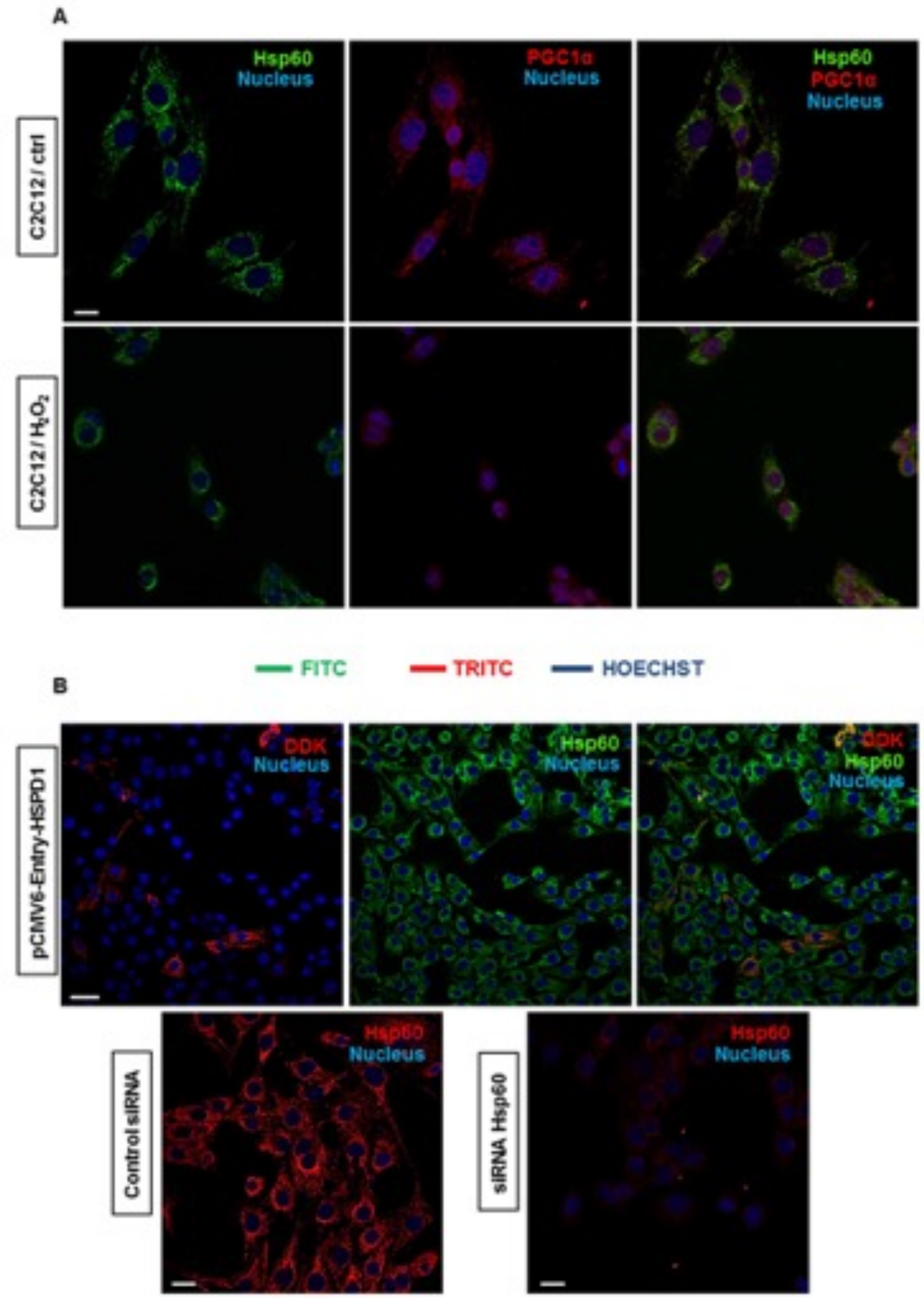

Figure S2. A, immunofluorescence images for Hsp60 and PGC1α of C2C12 myoblast cell line untreated (ctrl), and treated with H_2O_2, PGC1α translocated in the nucleus after 1 h treatment. Bar 25 µm. B, immunofluorescence images of C2C12 myoblasts cell line transfected with pCMV6-Entry-HSPD1 vector, the expression of the tag DDK demonstrated the efficiency of transfection of these cells (Bar 50 µm); and C2C12 myoblasts treated with Hsp60 siRNA (siRNA Hsp60) (Bar 25 µm).

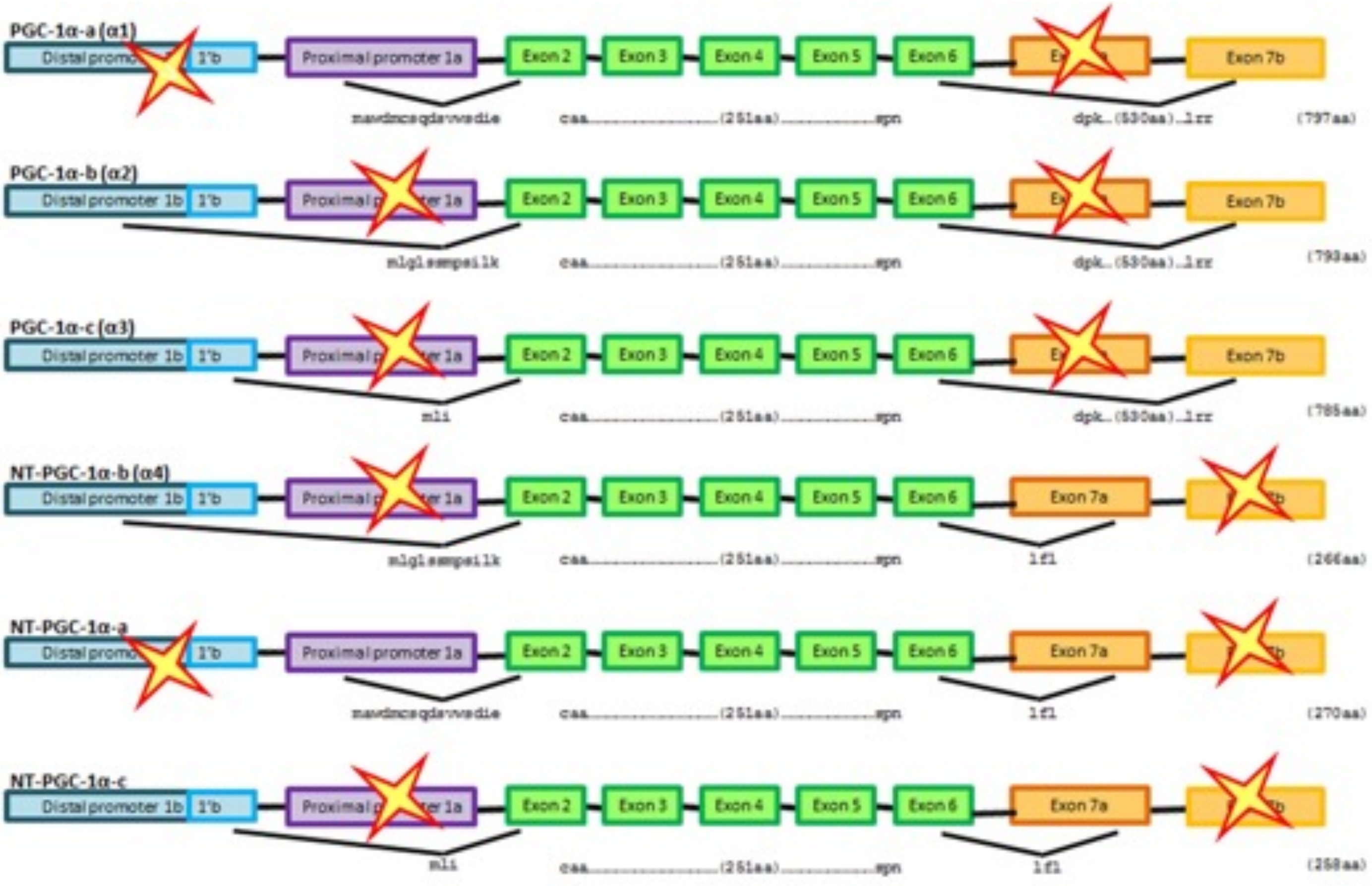

Figure S3. Schematic representation of PGC1α isoforms. See text for details.

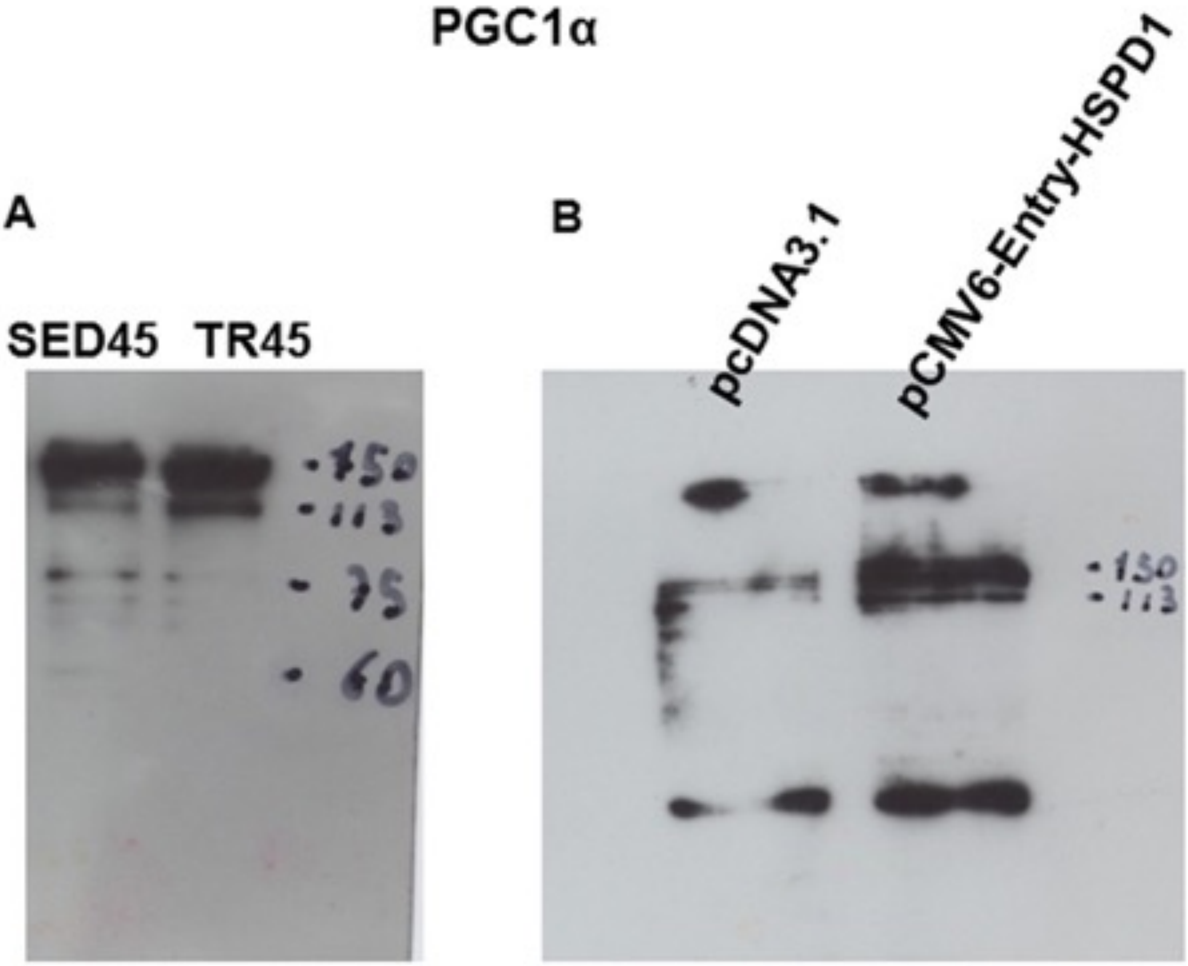

Figure S4. Full blots of Figure 6.

PGC-1α

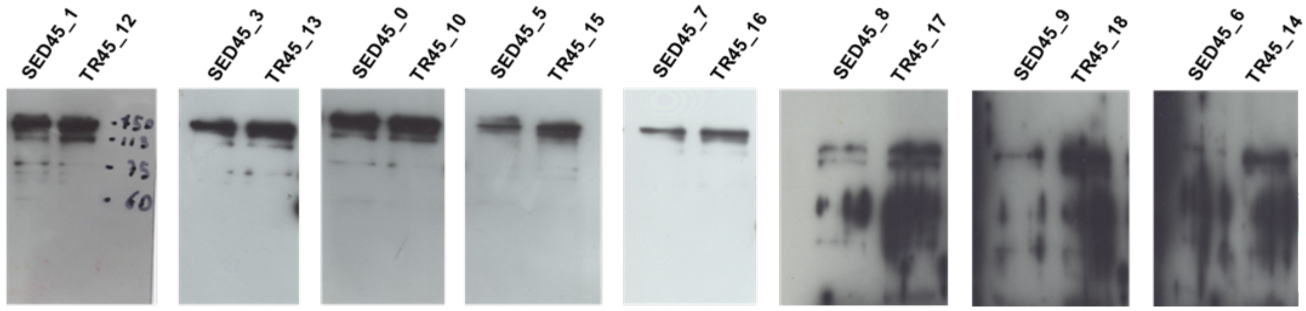

Full blots of the 8 mice used for the experiments in Figure 6.

Table S1
Primers used for semiquantitative qRT-PCR.

Primer	Target Sequence	Forward	Reverse
**BECN1_m**	template MGI databse ID OTTMUSG00000002791	5’-CGAGTGCCTTCATCCAAAAC-3’	5’-GTCCTGGCACCTCTCTAATG-3’
**mt-Cytb_m**	template MGI database ID 17711	5’-TAGCAATCGTTCACCTCCTC-3’	5’-TGTAGTTGTCTGGGTCTCCT-3’
**mt_12s_m**	template MGI database ID MGI:102493	5’-GATAAACCCCGCTCTACCTC-3’	5’-CATTGGCTACACCTTGACCT-3’
**PGC1 tot**	PubMed PMID: 23217713 (ref)	5’-TGATGTGAATGACTTGGATA-CAGACA-3’	5’-GCTCATTGTTGTACTGGTTGGATATG-3’
**PGC1 a1**	PubMed PMID: 23217713 (ref)	5’-GGACATGTGCAGCCAA-GACTCT-3’	5’-CACTTCAATCCACCCAGAAAGCT-3’
**PGC1 a2**	PubMed PMID: 23217713	5’-CCACCAGAATGAGTGA-CATGGA-3’	5’-GTTCAGCAAGATCTGGGCAAA-3’
**PGC1 a3**	PubMed PMID: 23217713	5’-AAGTGAGTAACCGGAGG-CATTC-3’	5’-TTCAGGAAGATCTGGGCAAAGA-3’
**PGC1 a4**	PubMed PMID: 23217713	5’-TCACACCAAACCCACA-GAAA-3’	5’-CAGTGTGTGTATGAGGGTTGG-3’
**HSP60_Mus**	MGI:MGI:96242	5’-ACGATCTATTGCCAAGGAGG-3’	5’-TCAGGGGTTGTCACAGGTTT-3’
**GADPH_Mus**	MGI:MGI:95640	5'-CAAGGACACTGAGCAAGA-GA-3'	5'-GCCCCTCCTGTTATTATGGG-3'
